# The trials and tribulations of linking real-world data: When research doesn’t go quite as planned

**DOI:** 10.23889/ijpds.v8i2.2328

**Published:** 2023-09-14

**Authors:** Helen Hodges

**Affiliations:** 1 CASCADE / Cardiff University, Cardiff, United Kingdom

## Objectives

Drawing upon learning from bringing youth justice data into SAIL Databank, this paper will focus some of the challenges associated with using real world data, and the implications that this for the research.

## Methods

As part of a feasibility study model how the probability of further offending changes over time for children looked after relative to their peers, two local authorities agreed to share historic risk assessment data. This was then linked to linking education, social services, health and family court data. The temporal coverage of the data was such that records did not go back to birth in many instances, and the chaotic lifestyles of some of the data subjects meant that it was not possible for an ALF to be created.

## Results

An examination of the match rates highlights where there may well be issues for other researchers wishing to understand the vulnerabilities and circumstances of those in the criminal justice system through larger datasets. Whilst the quality of the data provided placed limitations on the study, the exercise also demonstrated the potential opportunities afforded by data linkage to identify how best to support those with complex lives as they navigate the justice system. As one of the first Welsh studies, this study highlights the richness of the social science datasets available to researchers who are concerned with interface between justice and devolved policy areas.

## Conclusion

Whilst not all of the objectives of the study could be realised, the insights gained represent a methodological advancement, and contribute to understandings of the realities of working with real world data.

